# Single-nucleus RNA sequencing reveals HBV-driven metabolic reprogramming and TIMP1-mediated fibrosis in human-liver-chimeric mice

**DOI:** 10.3389/fcimb.2025.1654903

**Published:** 2025-09-02

**Authors:** Xiaonan Ren, Cong Wang, Boyin Qin, Hua Yang, Min Wu, Zhanqing Zhang, Wei Lu, Chao Wang, Yabin Liu, Xiaonan Zhang, Xiaohui Zhou

**Affiliations:** ^1^ Department of Laboratory Animal Science, Shanghai Public Health Clinical Center, Fudan University, Shanghai, China; ^2^ Department of Basic Scientific Research, Shanghai Public Health Clinical Center, Fudan University, Shanghai, China; ^3^ Department of Hepatology, Shanghai Public Health Clinical Center, Fudan University, Shanghai, China; ^4^ Centre for Research in Therapeutic Solutions, Biomedical Sciences, Faculty of Science and Technology, University of Canberra, Canberra, ACT, Australia

**Keywords:** HBV, snRNA-seq, liver humanized mice, TIMP1, ALB, URG

## Abstract

**Introduction:**

Hepatitis B virus (HBV) infection remains a leading cause of chronic liver disease, cirrhosis, and hepatocellular carcinoma worldwide. Despite advances in antiviral therapies, the mechanisms underlying HBV-induced metabolic reprogramming and liver fibrosis remain poorly understood.

**Methods:**

We employed single-nucleus RNA sequencing (snRNA-seq) which is particularly suitable for hepatocytic sequencing to dissect the transcriptional landscape of HBV-infected and uninfected hepatocytes in humanized URG mice (Hu-URG).

**Results and Discussion:**

Chronic HBV infection was successfully established in Hu-URG mice, with progressive increases in serum HBV DNA, HBsAg, and HBeAg levels. snRNA-seq revealed distinct human hepatocyte clusters (clusters 9, 16, 23) characterizing elevated expression of metabolic genes (*ALB, UGT2B17, CYP2A6*) in HBV-infected cells, while HBV-uninfected cells exhibited upregulation of TIMP1 and pro-fibrotic pathways. Immunofluorescence and histological analyses confirmed that HBV-uninfected hepatocytes (HBsAg^-^) displayed higher TIMP1 expression and reduced albumin (hALB) levels, correlating with increased collagen deposition in HBV-hu-URG mice. Notably, this TIMP1^+^HBsAg^-^hALB^low^ phenotype was also observed in liver biopsies from chronic HBV patients, underscoring its clinical relevance. Our findings highlight HBV-driven metabolic adaptation and identify TIMP1 as a potential mediator of fibrosis in uninfected hepatocytes, offering novel insights into HBV pathogenesis and therapeutic targeting.

## 1 Introduction

Chronic hepatitis B virus (HBV) infection continues to impose a substantial global health burden which affects over 296 million individuals worldwide (Cui et al., 2023). It is intricately linked to the development of viral hepatitis, liver cirrhosis, and hepatocellular carcinoma, posing a substantial threat to public health. Despite the therapeutic advancements, including nucleos(t)ide analogs and interferon-based regimens that suppress viral replication, functional cure rates remain a challenge (Lok, 2024), and the molecular interplay between HBV and host hepatocytes remains incompletely defined. Emerging evidence highlights the pivotal role of HBV-mediated metabolic reprogramming in viral persistence and immune evasion (Zhou et al., 2021). HBV infection is associated with widespread metabolic dysregulation, encompassing lipid, bile acid, glucose, amino acid, and nucleic acid metabolism (Diaz et al., 2022; Wang and Zhang, 2023).

Hepatocytes, the principal site of HBV replication, govern essential metabolic processes such as xenobiotic detoxification, lipid homeostasis, and bile acid synthesis. Due to their sensitivity to tissue dissociation, they have been poorly represented in current single-cell transcriptomic datasets. Although optimized dissociation protocols have improved hepatocyte viability, these remain predominantly applied to healthy liver tissues, leaving disease-specific adaptations underexplored. Moreover, tissue dissociation for single-cell RNA sequencing (scRNA-seq) introduces cell representation biases and *de novo* transcriptional stress responses, potentially masking the underlying biological state (Denisenko et al., 2020; Carlessi et al., 2023). In contrast, Single-nucleus RNA sequencing (snRNA-seq) bypasses the cell dissociation step by using detergents to release nuclei from intact cells, minimizing potential changes in gene expression resulting from enzymatic cell separation methods (Wu et al., 2019). When comparing the two platforms, they exhibit comparable diversity but distinct proportions of cell types in matched tissues. Notably, the proportion of hepatocytes in the liver is relatively higher in snRNA-seq data (Oh et al., 2022). Some studies have shown that snRNA-seq may be sufficient to replace scRNA-seq in certain scenarios and performs better in hepatocyte sequencing (Wen et al., 2022). Moreover, snRNA-seq has unique advantages in characterizing pre-mRNAs and long non-coding RNAs (lncRNAs) within nuclei (Zeng et al., 2016; Gao et al., 2017).

Recent studies have highlighted the role of host cellular metabolism in supporting HBV replication. For instance, HBV manipulates cellular metabolic pathways to enhance viral persistence and replication. Additionally, the expression of tissue inhibitors of metalloproteinases (TIMPs), such as TIMP1, has been implicated in liver fibrosis progression (Arthur, 2000; Li et al., 2020; Masuzaki et al., 2020). However, the metabolic and fibrogenic consequences of HBV infection at single-cell resolution remain elusive.

In this study, we integrated snRNA-seq with a humanized Tet-uPA^Tg^ Rag2^null^ Il2rg^null^ (URG) (Xie et al., 2019) mouse model, a system that supports engraftment of functional human hepatocytes to enable *in vivo* investigations of HBV infection, to characterize the transcriptional landscapes of HBV-infected versus uninfected bystander hepatocytes. Our findings reveal HBV-induced upregulation of peroxisomal and lipid metabolic genes, alongside a TIMP1-driven profibrotic signature in uninfected hepatocytes. These findings were validated in liver tissues from human HBV patients, bridging murine models to clinical pathology. By mapping HBV-host metabolic crosstalk and fibrosis drivers, this study provides a high-resolution atlas of HBV-host interactions and identifies TIMP1 as a key regulator of fibrosis in the HBV microenvironment.

## 2 Materials and methods

### 2.1 Mice

URG and liver humanized URG mice (Hu-URG) were obtained from the Beijing Vitalstar Biotechnology Co., Ltd. All animal experiments were performed in accordance with the procedures approved by the Institutional Animal Care and Use Committee of Shanghai Public Health Clinical Center (2020-A025-01).

### 2.2 Human liver samples

Human liver biopsies from HBV patient were collected with ethical approval from Shanghai Public Health Clinical Center (2018-S033-02), following written informed consent from the patients. The clinical characteristics of the patient are detailed in **Table 1**.

**Table 1:** The clinical information of the HBV patient.

G stage	S stage	Liver HBsAg	Liver HBcAg	HBsAg (IU/mL)	HBsAb (mIU/mL)	HBeAg (S/CO)	HBeA (S/CO)	HBeA (S/CO)	HBV DNA (IU/mL)	ALT (U/L)	AST (U/L)
G3	S3-4	+++	+	12584.6	-0.69	-0.246	0.02	13.19	15800000	578	555

Based on the immunohistochemical staining results of HBsAg or HBcAg in liver biopsy specimens from hepatitis B patients, the positivity is graded as follows: 0 to 4+ scale, corresponding to positivity in 0, 1-10, 11-25, 25-50% and more than 50% of the examined hepatocytes.

### 2.3 Blood sample collection and analyses

Mouse blood samples were collected at 0, 7, 13, 21, 30, 36, 43, 49, 57, 64, 70, 78, 85, and 91 days post inoculation (d.p.i.) (n=4 mice per time point). The samples were centrifuged at 600×g for 15 minutes, and the serum was separated and stored at −80°C for subsequent analyses. Serum human albumin (hALB), alanine aminotransferase (ALT), HBV DNA, hepatitis B surface antigen (HBsAg), and hepatitis B e antigen (HBeAg) levels were analyzed.

### 2.4 Human albumin ELISA

Serum hALB levels were measured using the Human Albumin ELISA Kit (Bethyl, Catalog E88-129) according to the manufacturer’s protocol.

### 2.5 Measurement of transaminase activity

Serum ALT activity was measured using the Roche Cobas C702 platform, following the manufacturer’s instructions (Roche modular, Basel, Switzerland).

### 2.6 HBV infection of humanized mice

At 56 days post transplantation, Hu-URG mice were intravenously inoculated with 100 µl of 8×10^8^ HBV genome equivalents (genotype D), resulting in HBV-infected Hu-URG mice (HBV-hu-URG). HBV particles were harvested from HepAD38 cell supernatants after doxycycline withdrawal. Specifically, cells were expanded and supernatants collected every 3–4 days. Clarified supernatants were then concentrated using 100-kDa Millipore centrifugal filters (3,220×g, 40 minutes), aliquoted, and stored at –80°C. Viral titers quantified by TaqMan PCR averaged 8×10^9^ copies/mL. Blood samples were collected at 7, 13, 21, 30, 36, 43, 49, 57, 64, 70, 78, 85, and 91 d.p.i. (n=2 mice per time point). The samples were processed as previously described, and the mice were subsequently sacrificed for the collection of liver and serum samples.

### 2.7 Quantification of serum HBV markers

Serum HBV DNA was quantified using the ABI Prism 7500 Sequence Detector system with TaqMan PCR Reagents (KHB, Shanghai, China). Serum levels of HBsAg and HBeAg were measured using the AutoLumo A2000PLUS platforms, following the manufacturer’s protocol.

### 2.8 Hematoxylin and eosin and Picrosirius Red staining

At the time of sacrifice, livers from URG, Hu-URG, and HBV-Hu-URG mice were fixed with 4% paraformaldehyde (PFA) for 24 hours, rinsed, and stored in 70% ethanol. Formalin-fixed specimens were embedded in paraffin, sectioned, and routinely stained with hematoxylin and eosin (H&E). For Picrosirius Red staining, a staining solution was prepared using Sirius Red and saturated picric acid. Rehydrated sections were stained in Picrosirius Red solution for 30 minutes. Quantification of Sirius Red-positive areas was performed using Image J software.

### 2.9 Immunohistochemical staining

At the time of sacrifice, livers from Hu-URG and HBV-hu-URG mice were fixed with 4% PFA for 24 hours, then dehydrated in 70% ethanol until paraffin processing. Tissues was cryosectioned into 5 μm sections and stained with antibody against hALB (1:2000), HBsAg (1:200), and Hepatitis B core antigen (HBcAg) (1:500).

### 2.10 Immunofluorescence staining

Mice liver tissues were embedded in OCT and frozen at -80°C for subsequent use. Tissues were cryosectioned into 8 µm sections. Chronic HBV patient liver tissues were fixed with 4% PFA, embedded in paraffin, and cryosectioned into 5 μm sections. After deparaffinizing, antigen retrieval was performed by boiling the samples in citrate buffer for 20 minutes in a microwave oven. Both frozen mouse sections and formalin-fixed paraffin-embedded (FFPE) patient sections were permeabilized in 1×PBS with 0.5% Triton X-100 for 30 minutes. The tissues were then blocked in 1×PBS with 3% BSA for 2 hours at room temperature. Primary antibodies were diluted in 1×PBS with 3% BSA and incubated overnight tin a wet chamber at 4°C: Rabbit anti-human TIMP1 (hTIMP1) (1:200) and Mouse anti-HBsAg (1:100)/Mouse anti-HBcAg (1:100); Rabbit anti-hTIMP1 (1:200)/Rabbit anti-Ki67 (1:600) and Goat anti-hALB (1:2000). Sections were washed three times with PBS containing 0.5% Tween 20 for 5 minutes each time. Secondary antibodies, Alexa Fluor 488 and Alexa Fluor 594; Alexa Fluor 568 and FITC, were diluted 1:1000 in PBS with 3% BSA and incubated for 1 hour at room temperature. Nuclei were stained with Hoechst. Imaging was performed using a Leica confocal microscope STELLARIS 8.

### 2.11 Nucleus isolation

Fresh liver samples were collected from mice, and nuclei were extracted. Chopped liver tissues were resuspended in 2 mL chilled nuclei lysis buffer. The homogenates were sequentially filtered through a 40 µm cell strainer and centrifuged at 4˚C, 500 × g for 5 minutes to precipitate the nuclei. The precipitate was resuspended in 200 µl cold PBS and stained with Trypan Blue.

### 2.12 Single-nucleus RNA sequencing

snRNA-seq was conducted by Neo-Biotechnology Co., Ltd. (Shanghai, China). Libraries for snRNA-seq were generated using the Chromium Single Cell 3’ library from 10×Genomics, following the manufacturer’s instructions. Approximately 18,000-20,000 nuclei were profiled per library. The raw sequence data have been deposited in the Genome Sequence Archive in National Genomics Data Center, China National Center for Bioinformation/Beijing Institute of Genomics, Chinese Academy of Sciences (GSA: CRA025748).

### 2.13 Data analysis

#### 2.13.1 General statistical analyses

Statistical analyses were performed using GraphPad Prism software. Group comparisons were made using unpaired Student’s t-tests. The number of repetitions is indicated in the legends of each graph. Results were expressed as means ± SEM. A *P*-value < 0.05 was considered statistically significant. snRNA-seq data processing, statistical analyses, and visualization were performed using the R programming language (version 4.1.3).

#### 2.13.2 snRNA-seq data processing and analysis

BCL files were demultiplexed and converted into FASTQ format using bcl2fastq utility from Illumina BaseSpace Sequence Hub. The FASTQ files were processed using Cell Ranger 7.1.0. Raw sequencing reads were aligned to the combined human and mouse reference genome (GRCh38 and GRCm39) using the corresponding transcriptome annotation files: Human GRCh38 (GENCODE v44/Ensembl 110 annotations) and Mouse GRCm39 (GENCODE vM33/Ensembl 110 annotations). The HBV-infected sample were aligned to the HBV D genome (AF411411.1 GI:15778332). The Raw gene-barcode matrices from Cell Ranger were used for downstream analysis in R using the Seurat package (version 4.3.0). Matrices were imported using the Read10×() and CreateSeuratObject() functions. Quality control filtering was performed independently for each sample using the following criteria: nFeature_RNA > 200, nFeature_RNA < 5000, nCount_RNA < 50000, and <12.5% mitochondrial genes. Gene expression data were normalized using the NormalizeData() function with the “LogNormalize” method. Data integration across samples was performed with FindIntegrationAnchors() and IntegrateData(). Clustering was done using FindClusters() (resolution = 0.5), and dimensional reduction was achieved via principle component analysis (PCA) and uniform manifold approximation and projection (UMAP). Differentially expressed genes (DEGs) were identified using FindMarkers(). Genes with *P* < 0.05 and an average absolute fold change > 1.5 were considered significant.

### 2.14 Identification of HBV-infected single cells

To identify HBV-infected single cells, we used STAR (v2.7.10b) to map all snRNA sequences from each sample to the HBV genome. Mapped reads were extracted using samtools (v1.16.1), and the corresponding barcode sequences were identified using seqkit (v2.3.0). The HBV read count for each barcode was calculated to distinguish HBV-infected and uninfected single cells for subsequent comparative analysis.

### 2.15 Hallmark pathway analysis

To explore pathway differences between HBV-infected and uninfected single-cell subpopulations, hallmark pathway analysis was performed using the Molecular Signatures Database (MSigDB) Hallmark gene sets. Gene Set Enrichment Analysis (GSEA) was performed using the GSEA() function from the clusterProfiler R package (v4.6.2), with default parameters. The Hallmark gene sets were obtained from the Molecular Signatures Database (MSigDB). Genes were ranked by average log_2_ fold change between different cell groups. Enrichment scores (ES), nominal P-values, and false discovery rate (FDR) q-values were calculated according to the standard GSEA procedure. Pathways with FDR < 0.25 and nominal *P* < 0.05 were considered significantly enriched.

## 3 Results

### 3.1 Human hepatocytes engraftment in URG mice

**Figure 1:**
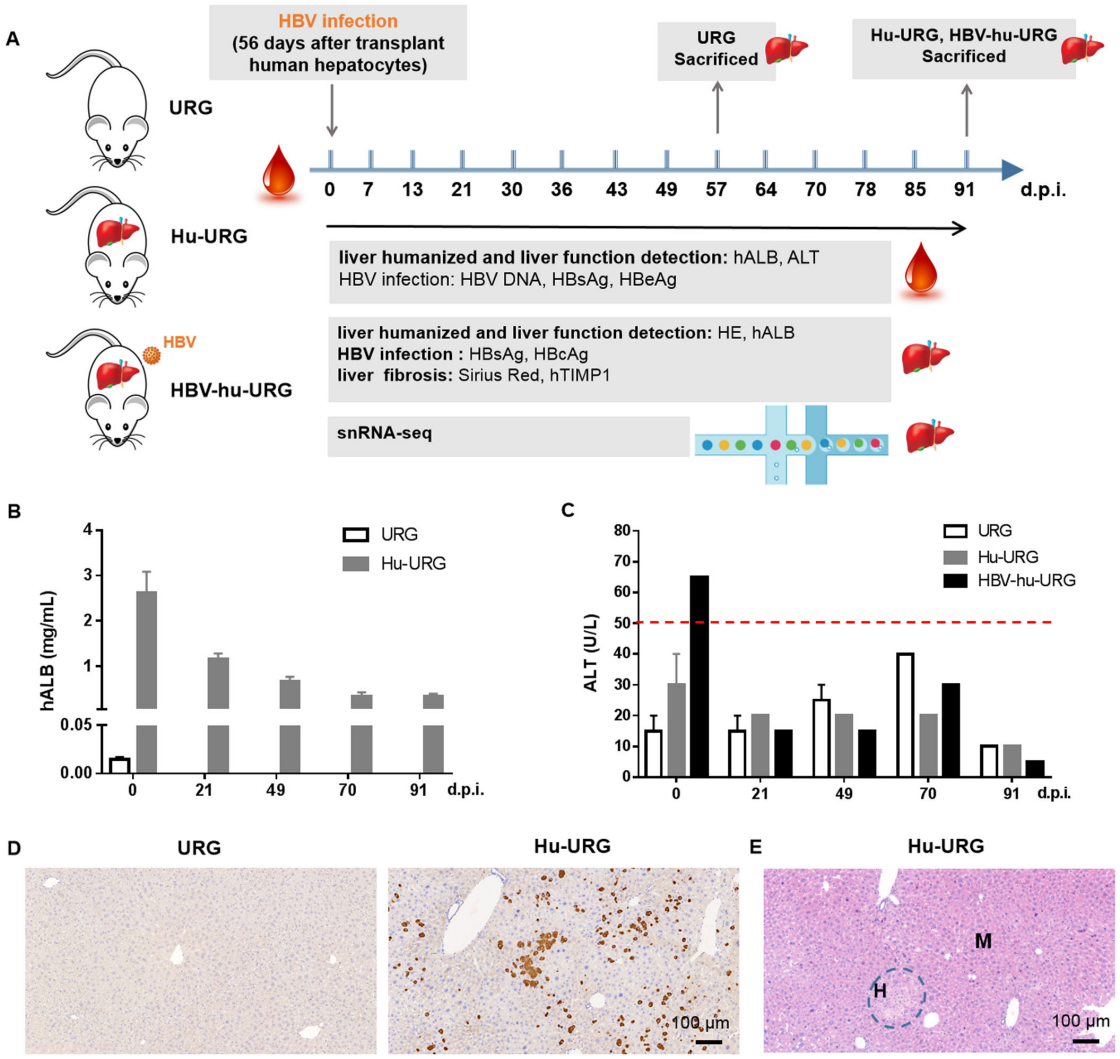
URG mice support engraftment of human hepatocytes in the liver. **(A)** Schematic representation of the experimental design and analysis. URG mice were transplanted with human hepatocytes to establish humanized liver mice, followed by HBV infection to generate HBV-infected mice. Serum samples were collected to assess liver humanization, liver function, and HBV infection. The mice were subsequently sacrificed, and liver sections were stained to evaluate liver humanization, liver function, HBV infection, and liver fibrosis. Liver tissue nuclei were extracted and used for snRNA-seq. **(B)** Serum hALB levels were measured in URG (n=2) and Hu-URG (n=4) mice at various d.p.i. Data are presented as the mean ± SEM. **(C)** Serum ALT levels were measured in URG (n=2), Hu-URG (n=2), and HBV-hu-URG (n=2) at various d.p.i. Data are presented as the mean ± SEM. **(D)** Representative liver samples from URG (n=2) and Hu-URG (n=4) were subjected to IHC staining for human albumin (Scale bar = 100 μm). **(E)** H&E staining of liver samples from Hu-URG mice. Human hepatocytes (H) and mouse hepatocytes (M) areas are indicated (Scale bar = 100 μm).

The experimental design is illustrated in **Figure 1A**. URG mice were transplanted with human hepatocytes, resulting in serum hALB levels rising to 1.58-3.76 mg/mL at 0 d.p.i. (56 days post-transplantation) and then gradually declining (**Figure 1B**). No significant differences in the liver function biomarker ALT were observed among URG, Hu-URG, and HBV-hu-URG mice (**Figure 1C**). Immunohistochemical staining performed at 91 d.p.i. revealed that human hepatocytes in Hu-URG mice were positive for hALB (**Figure 1D**). Histological analysis further demonstrated that human hepatocytes (H) could be easily distinguished from mouse hepatocytes (M) based on their paler color and larger size (**Figure 1E**).

### 3.2 HBV infection in Hu-URG mice

**Figure 2:**
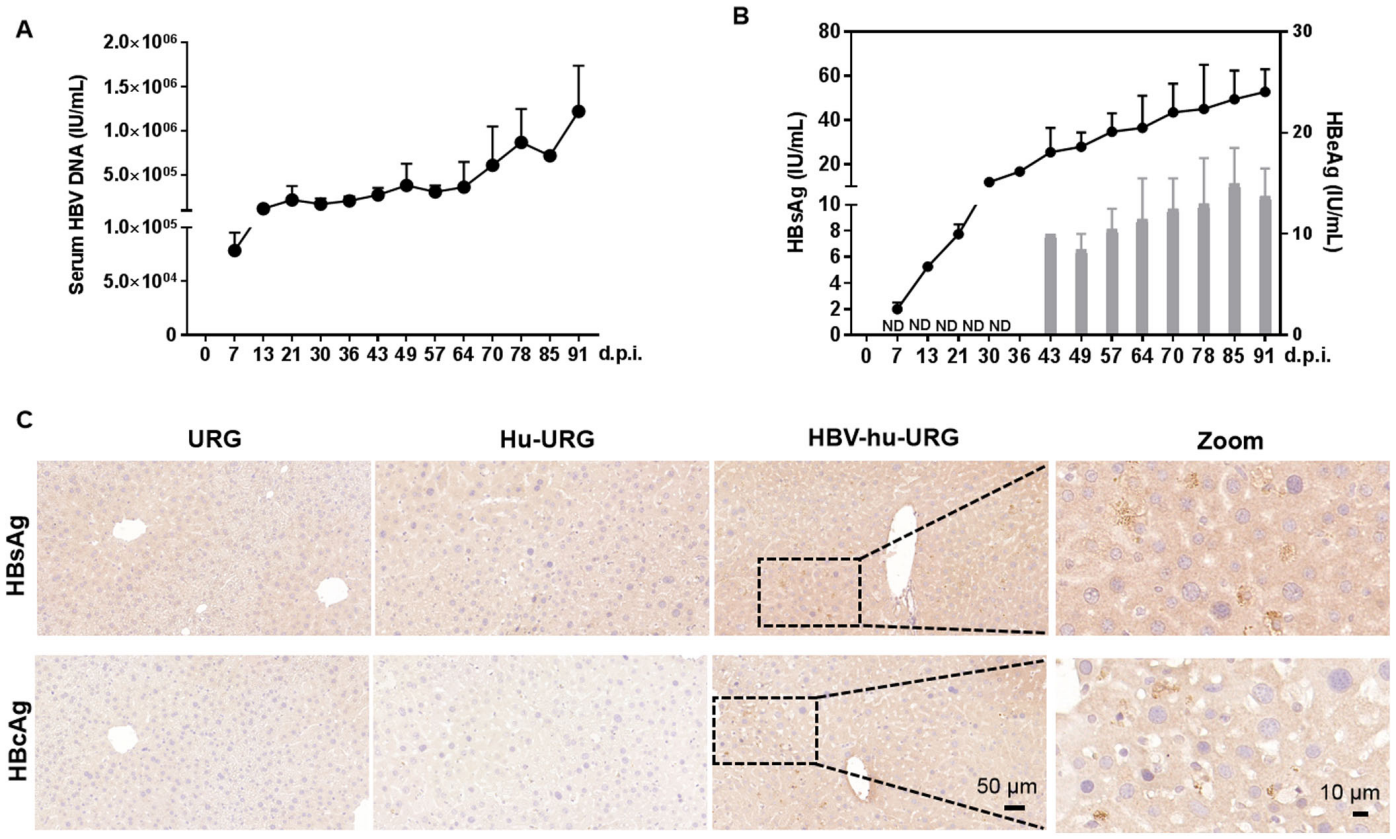
HBV infection in Hu-URG mice. Hu-URG (n=2) mice were inoculated with HBV (genotype D, 8×10^8^ HBV genome equivalents). Serum samples were collected at various d.p.i. Liver samples were collected at termination time points (57–91 d.p.i.). **(A)** Serum HBV genomic DNA was examined in HBV-infected Hu-URG mice at various time points post infection. Data are presented as the mean ± SEM. **(B)** HBsAg and HBeAg levels in serum were measured at various time points post infection. ND: not detectable. Data are presented as the mean ± SEM. **(C)** Representative liver samples from URG, Hu-URG and HBV-hu-URG mice were subjected to immunohistochemical staining for HBsAg and HBcAg (Scale bars = 50 μm and 10 μm, see images).

56 days after human hepatocyte transplantation (0 d.p.i.), Hu-URG mice were inoculated with 8×10^8^ HBV genome equivalents (genotype D) collected from HepAD38 supernatants. Chronic HBV infection was successfully established in all mice. HBV DNA was detected in serum samples from 7 to 91 d.p.i., with viral titers progressively increasing to 1.2×10^6^ IU/mL by 91 d.p.i. (**Figure 2A**). Serum HBsAg and HBeAg levels increased to 53 IU/mL and 13 IU/mL, respectively (**Figure 2B**). Liver samples collected from URG, Hu-URG, and HBV-Hu-URG mice sacrificed between 57 and 91 d.p.i. were analyzed. As shown in **Figure 2C**, HBsAg and HBcAg were expressed in the livers of HBV-Hu-URG mice.

### 3.3 Single-nucleus resolution identifies and distinguishes HBV-induced differences in human and mouse cells

**Figure 3:**
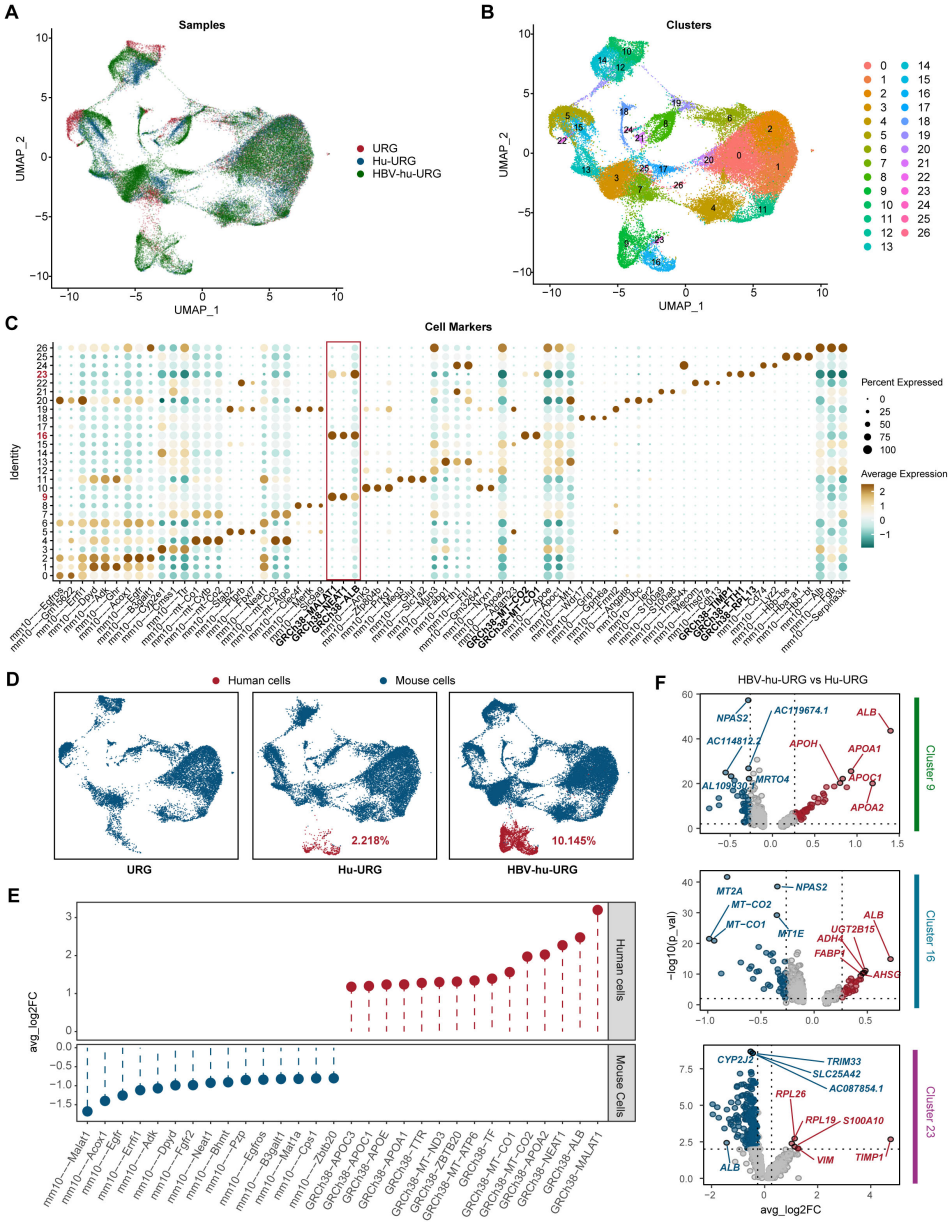
snRNA-seq identifies human and mouse populations in URG, Hu-URG and HBV-hu-URG mouse livers. **(A)** UMAP projection of combined snRNA-seq data from URG (red), Hu-URG (blue), and HBV-hu-URG (green). Each dot represents a single nucleus, and the clustering pattern reflects transcriptional similarities between cells from different groups. **(B)** UMAP plot showing clustering analysis of snRNA-seq data. Each cluster represents a transcriptionally distinct cell population. **(C)** Dot plot showing expression patterns of cell markers across the identified clusters. The x-axis lists marker genes, while the y-axis shows cluster identity. The size of each dot reflects the percentage of cells within the cluster expressing the gene, and the color intensity represents the average expression level. The red box highlights human-specific markers, which are predominantly enriched in human hepatocyte clusters. **(D)** UMAP plots showing the proportion of human and mouse cells in URG, Hu-URG, and HBV-hu-URG samples. Human cells (red) and mouse cells (blue) are shown. **(E)** Differential expression analysis between human and mouse cells showing genes with significant upregulation in human (red) and mouse (blue) hepatocytes. **(F)** Volcano plots showing DEGs in human hepatocytes between HBV-hu-URG and Hu-URG samples across clusters 9, 16, and 23. Red dots represent significantly upregulated genes, and blue dots represent downregulated genes.

To investigate the pathogenic mechanisms underlying HBV infection progression, we performed snRNA-seq analysis on liver samples from URG, Hu-URG, and HBV-hu-URG mice. A total of 55,398 single nuclei were obtained (11,838 from URG, 16,680 from Hu-URG, and 26,880 from HBV-hu-URG). UMAP visualization of the combined data revealed 27 distinct cell clusters (**Figures 3A, B**). Clustering analysis successfully identified human and mouse hepatocyte populations based on species-specific gene expression patterns. Specifically, clusters 9, 16, and 23 were classified as human hepatocytes, characterized by the high expression of human genes such as *MALAT1*, *NEAT1*, *ALB*, *TIMP1*, *FTH1*, *RPL13*, as well as mitochondrial genes involved in oxidative phosphorylation. In contrast, other clusters exhibited high expression of mouse-specific genes, including *Alb*, *Apoe*, *Apoa2*, and *Ttr*, indicating that these clusters represented mouse hepatocytes (**Figure 3C**). The proportions of human cells in Hu-URG and HBV-Hu-URG mice were 2.218% and 10.145%, respectively (**Figure 3D**), demonstrating successful engraftment and a notable expansion of human hepatocytes following HBV infection.

To further explore the impact of HBV infection at the single-cell level, we first compared gene expression between human and mouse hepatocytes to establish species-specific transcriptional profiles. Human cells exhibited significantly higher expression of *MALAT1*, *ALB*, *NEAT1*, and *APOA2*, consistent with their role in human-specific liver functions. In contrast, mouse cells exhibited high expression of *Malat1*, *Acox1*, *Egfr*, *Errfi1*, and *Adk*, confirming expected species-specific differences (**Figure 3E**). We then compared gene expression within human cells (clusters 9, 16, and 23) between Hu-URG and HBV-hu-URG mice. In clusters 9 and 16, genes involved in lipid metabolism and liver function, particularly *ALB*, were significantly upregulated in HBV-infected cells, suggesting that HBV infection promotes metabolic reprogramming and adaptation. In contrast, cluster 23 exhibited a distinct transcriptional profile characterized by low expression of *ALB* and elevated expression of pro-fibrotic and inflammatory genes, including *TIMP1* and *S100A10*. This unique expression pattern in cluster 23 indicates a shift toward a fibrosis-associated state, distinct from the metabolic adaptation observed in clusters 9 and 16. The increased expression of *TIMP1* is particularly notable, as *TIMP1* is a key regulator of extracellular matrix remodeling and liver fibrosis progression. These findings demonstrate that snRNA-seq can effectively distinguish human and mouse cell populations, define HBV-induced transcriptional changes at single-cell resolution, and uncover distinct metabolic and fibrotic responses in HBV-infected and bystander human cells. Key genes such as *ALB* and *TIMP1* emerged as central regulators of HBV-associated metabolic reprogramming and fibrosis, highlighting potential therapeutic targets for HBV-related liver disease (**Figure 3F**).

### 3.4 Cluster-specific metabolic and fibrotic responses in HBV-infected and uninfected Cells

**Figure 4:**
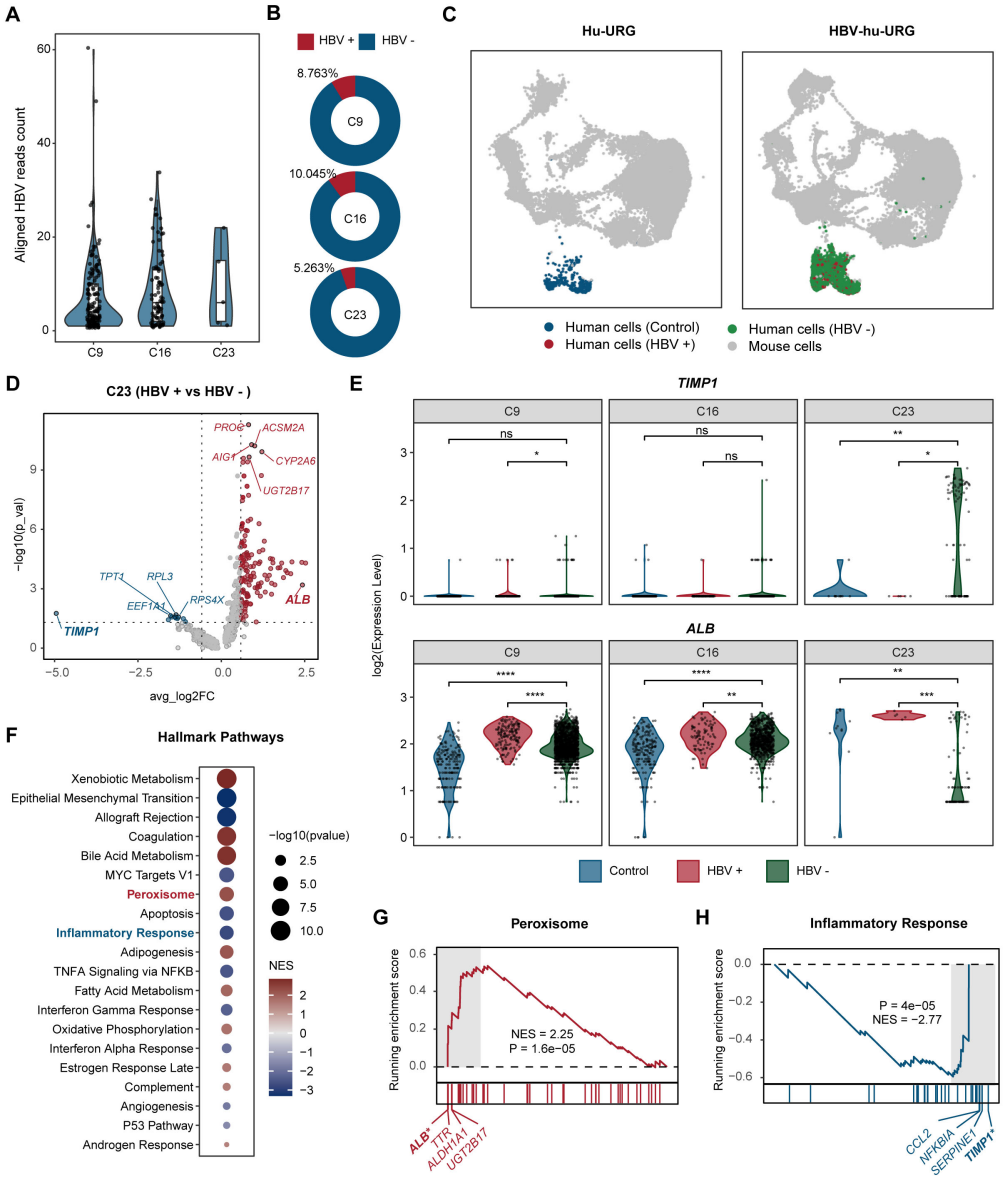
Comparative analysis of HBV-infected and uninfected human hepatocytes. **(A)** Aligned HBV read counts in clusters 9, 16, and 23 in HBV-hu-URG mice. Each dot represents a single cell. HBV read counts are significantly lower in cluster 23, suggesting a lower rate of HBV infection in this cluster. **(B)** Proportion of HBV-infected cells in clusters 9, 16, and 23 in HBV- hu-URG mice. **(C)** UMAP projection showing four distinct cell populations in Hu-URG and HBV- hu-URG, including mouse cells (gray dots), control human cells (blue dots), HBV-uninfected human cells (HBV^-^, green dots), and HBV-infected human cells (HBV^+^, red dots). **(D)** Volcano plot of differentially expressed genes between HBV^+^ and HBV^-^ cells in cluster 23 of HBV-hu-URG mice. Genes upregulated or downregulated by more than 1.5-fold are shown in red and blue, respectively. **(E)** Violin plots showing *TIMP1* and *ALB* expression in clusters 9, 16, and 23 among control (blue), HBV-infected (red), and HBV-uninfected (green) cells. **(F)** Hallmark pathway enrichment analysis of differentially expressed genes between HBV^+^ and HBV^-^ cells in cluster 23. Dot size indicates -log10 (p-value), and color indicates normalized enrichment score (NES). **(G)** GSEA plot for peroxisome pathway. **(H)** GSEA plot for inflammatory response pathway. The p-value was calculated by the Wilcoxon test. ns, not significant, *p < 0.05, **p < 0.01, ***p < 0.001, and ****p < 0.0001.

To investigate the transcriptional differences induced by HBV infection at the single-cell level, we first distinguished HBV-infected and uninfected cells by mapping sequencing reads to the HBV genome. We found that cluster 23 exhibited a lower HBV read count compared to clusters 9 and 16 in HBV-hu-URG mice, indicating that although this cluster contained both HBV-infected and uninfected cells, HBV-uninfected cells predominated. (**Figure 4A**). Further analysis revealed that the proportion of HBV-infected cells in cluster 23 was only 5.263%, which was significantly lower than in cluster 9 (8.763%) and cluster 16 (10.045%) (**Figure 4B**). UMAP plots further revealed four distinct clusters in the livers of Hu-URG and HBV-hu-URG mice (**Figure 4C**). Given the distinct behavior of cluster 23, we further analyzed transcriptional differences between HBV-infected and uninfected cells within this cluster. Genes significantly upregulated in HBV-infected cells of cluster 23 were primarily related to liver metabolic enzymes, including *ALB*, *UGT2B17*, *CYP2A6*, *ACSM2A*, *AIG1*, and *PROE*. In contrast, genes such as *TIMP1*, *TPT1*, *EEF1A1*, *RPL3*, and *RPS4X* were significantly more highly expressed in HBV-uninfected cells of cluster 23 (**Figure 4D**). Previous research has demonstrated that the abundance of most metabolic intermediates downstream of glucose is elevated during HBV infection (Zhou et al., 2021). Notably, *TIMP1* was significantly upregulated in HBV-uninfected cells of cluster 23, while *ALB* was notably highly expressed in HBV-infected cells across clusters 9, 16, and 23 (**Figure 4E**).

To better understand the functional implications of these transcriptional differences, we performed hallmark pathway analysis on differentially expressed genes in HBV-infected and uninfected cells within cluster 23. Genes involved in peroxisome, bile acid metabolism, and adipogenesis were enriched in HBV-infected cells of cluster 23. In contrast, genes related to the inflammatory response and epithelial-mesenchymal transition were more prominent in HBV-uninfected cells of cluster 23, suggesting that these cells may adopt a pro-fibrotic and inflammatory phenotype, potentially triggered by a paracrine or bystander effect from neighboring HBV-infected cells of cluster 23 (**Figure 4F**). Specifically, peroxisome pathway showed marked activation (NES= 2.25, *P*=1.6 e-05) and the related genes such as *ALB*, *TTR*, *ALDH1A*, and *UGT2B17* were upregulated in HBV-infected cells of cluster 23 (**Figure 4G**). Conversely, inflammatory response pathways were strongly suppressed (NES=-2.77, *P*=4e-05) and the genes like *TIMP1*, *SERPINE1*, *NFKBIA*, and *CCL2* were more highly expressed in HBV-uninfected cells of cluster 23 (**Figure 4H**).

These findings suggest that HBV infection enhances cellular metabolism to support viral replication, while neighboring uninfected cells respond by activating fibrotic and inflammatory pathways. The contrasting transcriptional states of HBV-infected and uninfected cells in cluster 23 highlight the complex host-virus interactions that may drive HBV-associated liver fibrosis and metabolic reprogramming.

### 3.5 hTIMP^+^ HBsAg^-^ hALB^low^ human hepatocytes in HBV-hu-URG mouse after HBV infection and liver fibrosis

**Figure 5:**
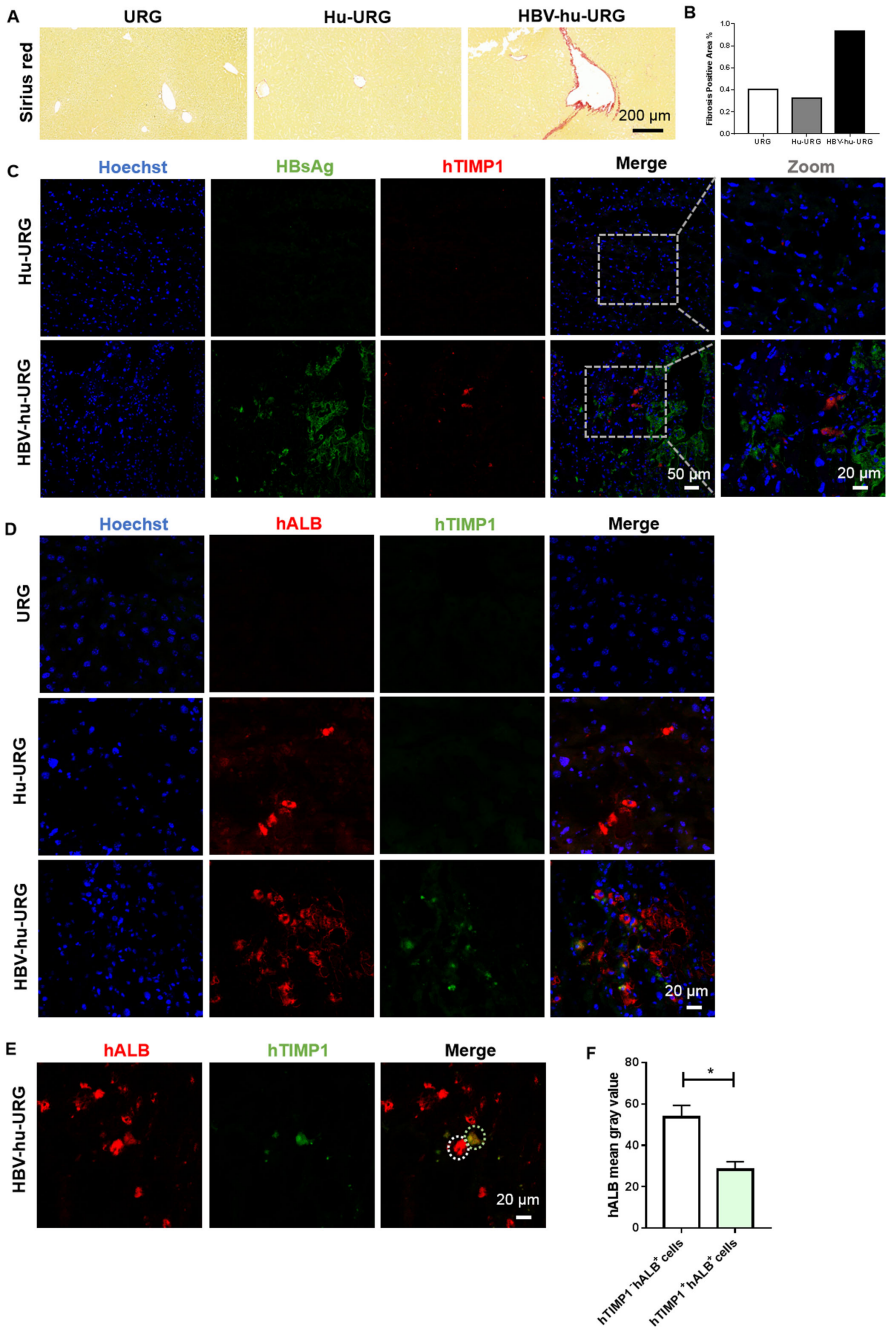
Picrosirius Red staining of liver fibrosis and IF staining verified hTIMP1^+^HBsAg^-^ hALB^low^ human hepatocytes in HBV-hu-URG mice. **(A)** Picrosirius Red staining of liver samples from URG, Hu-URG, and HBV-hu-URG mice (Scale bar = 200 μm). **(B)** Analysis of fibrosis area using Image (J) **(C)** IF images of hTIMP1(red), HBsAg (green), and Hoechst (blue) in Hu-URG and HBV-hu-URG mice (Scale bars = 50 μm and 20 μm, see images). **(D)** IF images of hTIMP1 (green), hALB (red) and Hoechst (blue) in URG, Hu-URG, and HBV-hu-URG mice (Scale bar = 20 μm). **(E)** IF images of hTIMP1 (green) and hALB (red) in HBV-hu-URG mice. White circle: hTIMP1^-^ hALB^+^ cell; green circle: hTIMP1^+^ hALB^+^ cell (Scale bar = 20 μm). **(F)** hALB levels of hTIMP1^-^ hALB^+^ cells (n= 6) and hTIMP1^+^ hALB^+^ cells (n= 4) analyzed using Image (J) Data are presented as the mean ± SEM. *p<0.05.

To validate the snRNA-seq findings, liver samples from URG, Hu-URG, and HBV-hu-URG mice sacrificed 57–91 d.p.i. were analyzed using picrosirius red staining to assess liver fibrosis. Compared to URG and Hu-URG mice, HBV-hu-URG mice exhibited significant collagen accumulation (**Figure 5A**). Image J analysis revealed that the percentage of fibrosis-positive areas was 0.405% in URG mice, 0.326% in Hu-URG mice, and 0.931% in HBV-Hu-URG mice (**Figure 5B**). Multiple immunofluorescence analysis of Hu-URG and HBV-hu-URG mouse livers showed that HBV-uninfected (HBsAg^-^) cells had significantly higher expression of hTIMP1(**Figure 5C**). These cells were confirmed to be human hepatocytes, as indicated by the hepatocyte marker hALB (hALB^+^) (**Figure 5D**). Notably, these HBsAg^-^ hTIMP1^+^ hALB^+^ hepatocytes exhibited significantly lower expression of hALB (hALB^low^) (**Figures 5E, F**), suggesting that HBV-uninfected human hepatocytes are more likely to express hTIMP1, potentially contributing to liver fibrosis, while simultaneously reducing hALB expression. Through Ki67 immunofluorescence analysis, we further revealed that the proliferative capacity of HBV-infected human hepatocytes was higher than that in the uninfected Hu-URG group (**Supplementary Figure 1**).

### 3.6 hTIMP^+^HBsAg^-^ hepatocytes in chronic HBV patient

**Figure 6:**
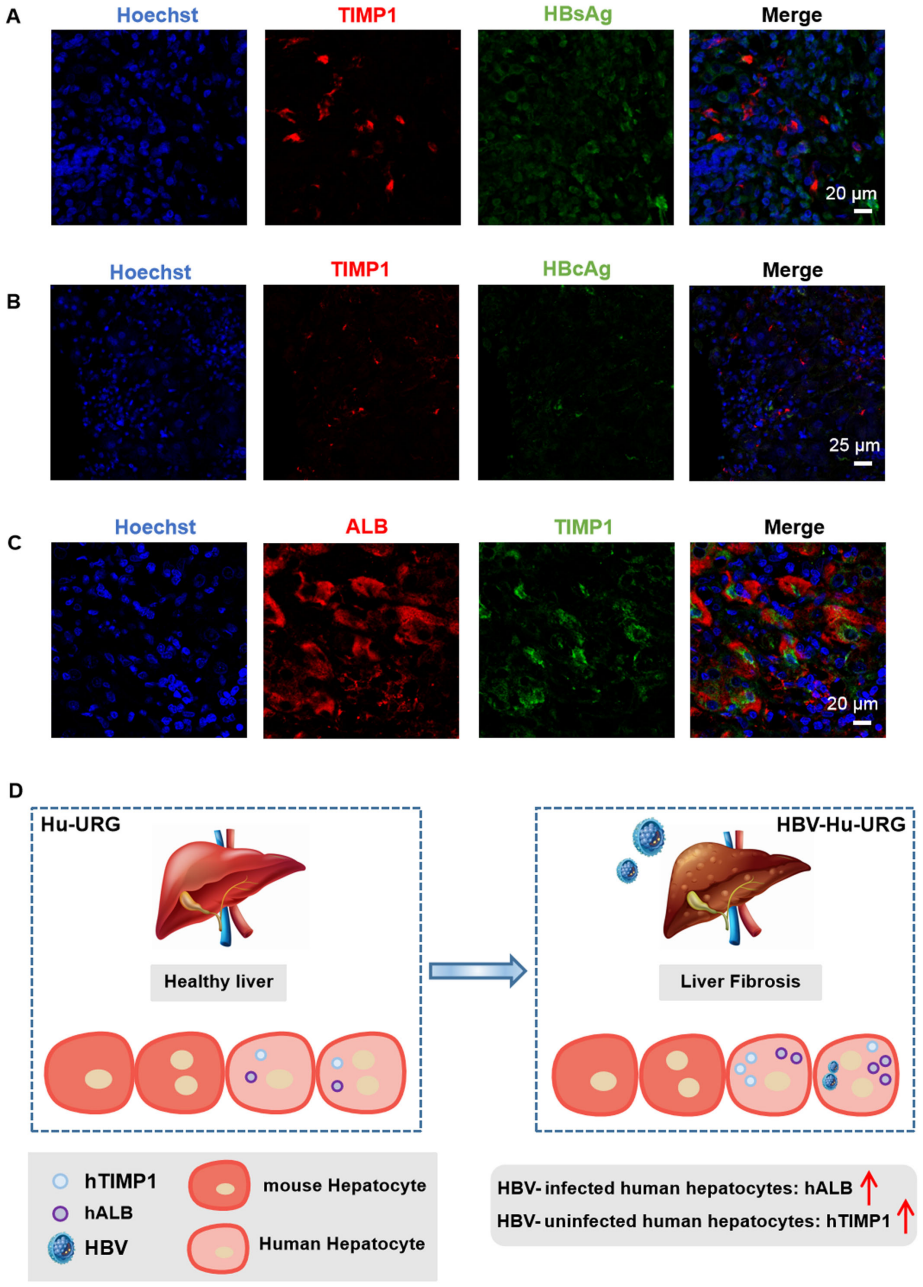
IF staining verified hTIMP1^+^ HBsAg^-^ hepatocytes in HBV patient. **(A)** IF images of hTIMP1 (red), HBsAg (green), and Hoechst (blue) (Scale bar = 20 μm). **(B)** IF images of hTIMP1 (red), HBcAg (green), and Hoechst (blue) (Scale bar = 25 μm). **(C)** IF images of hALB (red), hTIMP1 (green), and Hoechst (blue) (Scale bar = 20 μm). **(D)** Differential expression of hTIMP1 and hALB in HBV-uninfected and infected human hepatocytes.

To determine whether the observations in mice were relevant to human disease, we examined FFPE liver specimens from chronic HBV patients. Similar to the humanized mouse model, HBsAg^-^ or HBcAg^-^ cells in human liver tissue also exhibited significantly higher expression of hTIMP1 (**Figures 6A, B**). These cells were confirmed to be hepatocytes (hALB^+^) (**Figure 6C**). We identified hTIMP1-positive and HBsAg-negative hepatocytes in the liver tissue of chronic HBV patient, indicating a similar pattern to that observed in the humanized mouse model. Together, these results illustrate that HBV-uninfected human hepatocytes exhibit high expression of hTIMP1, whereas HBV-infected human hepatocytes show high expression of hALB (**Figure 6D**).

## 4 Discussion

This study leverages the novel Hu-URG mouse model combined with snRNA-seq to elucidate the intricate interplay between HBV infection, host metabolic reprogramming, and fibrosis progression. The Hu-URG model, characterized by a relatively high chimerism rate (20-95%) through Tet-On system-driven inducible human hepatocyte repopulation, effectively bridges the gap between murine studies and human pathophysiology by replicating clinical HBV-infected liver tissue signatures (Song et al., 2009; Lai et al., 2021). It has been documented that snRNA-seq shown advantages and performs better than scRNA-seq in hepatocyte sequencing. The methodology of applying snRNA-seq in HBV infected Hu-URG mouse model provide the ability to distinguish between infected and bystander hepatocytes at single-cell resolution, which addresses a critical need in virology, where heterogeneous cellular responses often obscure mechanistic interpretations (Alvarez et al., 2022; Andrews et al., 2022).

Our findings revealed significant upregulation of metabolic enzymes such as *ALB, UGT2B17, CYP2A6*, and *ACSM2A* in HBV-infected hepatocytes, aligning with previous research on HBV-induced metabolic reprogramming. Specifically, HBV’s impact on lipid and glucose metabolism is evident through impaired triglyceride synthesis and reduced lipid droplet formation, as observed by Yasumoto (Yasumoto et al., 2017). Lamontagne (Lamontagne et al., 2018) further supports these findings, noting increased expression of long-chain fatty acids like myristate and palmitate in HBV-infected hepatocytes. The role of CD36 in HBV replication and subsequent fatty acid synthesis underscores this metabolic shift (Miquilena-Colina et al., 2011; Huang et al., 2017). Additionally, the upregulation of *ALB* and *UGT2B17* highlights enhanced detoxification processes and bile acid metabolism, crucial for lipid digestion, consistent with Wang’s (Wang et al., 2018)observation of elevated liver cholesterol levels in HBV infection. Notably, *UGT2B17*, essential for lipid-to-carbohydrate conversion, was significantly upregulated in infected cells, with UGT2B17-deficient livers showing increased abundance of altered proteins enriched in metabolic, chemical defense, and immune-related pathways (Rouleau et al., 2025). These findings illustrate HBV’s profound impact on hepatic metabolic networks, and support previous research shown that HBV functions as a “metabolic virus,” (Bar-Yishay et al., 2011)akin to HCV’s manipulation of lipid droplets for replication (Barba et al., 1997; Vogt et al., 2013).

HBV infection and uninfected hepatocytes exhibit significant spatial heterogeneity within the liver microenvironment. Infected cells create a favorable niche for viral persistence through metabolic reprogramming, such as enhanced lipid metabolism, whereas uninfected cells display pro-inflammatory and pro-fibrotic features, with notable upregulation of genes like *TIMP1, SERPINE1*, and *CCL2*, *TIMP1* plays a dual role in fibrosis progression: it inhibits extracellular matrix degradation by suppressing matrix metalloproteinases (MMPs) and activates hepatic stellate cells (HSCs) via the CD63 signaling pathway, driving collagen deposition (Zan et al., 2013; Naim et al., 2017), thereby promoting collagen accumulation and fibrogenesis. Histopathological validation in HBV-infected URG mice indeed confirmed increased collagen accumulation in these mice, correlating with higher TIMP1 expression in HBV-uninfected hepatocytes. Clinical studies indicate that serum TIMP1 levels in chronic HBV patients correlate positively with fibrosis stages, with diagnostic efficacy (AUROC>0.9) validated across multiple cohorts (Zhu et al., 2012; Zhang et al., 2021). The pro-fibrotic phenotype of uninfected hepatocytes may result from paracrine signals from infected cells or immune microenvironment remodeling. For instance, HBV X protein (HBx) induces neighboring hepatocytes to secrete TGF-β1, activating HSCs and promoting their proliferation and collagen synthesis (Guo et al., 2009; Park et al., 2015). Additionally, NKT cells and Kupffer cells in HBV infection models secrete pro-fibrotic factors like IL-13 and TGF-β1 (Jin et al., 2011; Song et al., 2021), exacerbating microenvironmental imbalance. The spatial segregation of metabolic pathways (e.g., PPAR-mediated lipid metabolism (Kim et al., 2007) and inflammatory signals between infected and uninfected regions underscores the challenge in halting fibrosis progression with current antiviral therapies.

This cellular heterogeneity may stem from HBV’s differential regulation of host transcription networks. HBx sustains lipid metabolic advantages in infected cells by activating metabolic-related transcription factors like PPAR and fosters a fibrotic microenvironment through the TGF-β1/OCT4/Nanog axis (Li et al., 2022). In HBV/HCV co-infection models, this pathway activation synergistically accelerates liver fibrosis. Moreover, some studies have suggested that hepatocytes may undergo epithelial-to-mesenchymal transition (EMT) to contribute to liver fibrosis (Zeisberg et al., 2007; Cicchini et al., 2014), the role of uninfected hepatocytes in this process remains undefined.

Identifying TIMP1 as a potential driver of liver fibrosis in HBV-infected mice highlights the complexity of host-virus interactions. While infected cells turn on supporting viral replication through metabolic reprogramming, neighboring uninfected bystander hepatocytes may promote fibrosis through elevated TIMP1 expression, which is independent of direct viral cytopathic effects. This dichotomy underscores the importance of understanding both infected and bystander cell responses in the context of viral hepatitis. This “metabolic-fibrotic” dual strategy not only aids the virus in evading immune clearance but also promotes chronic disease by remodeling the microenvironment. Although existing antiviral drugs can suppress viral replication, they fail to effectively disrupt the pathological crosstalk between infected and uninfected zones, highlighting the need for targeted microenvironmental regulation therapies.

Despite these insights, several questions remain. The Hu-URG model lacks functional immune components, precluding analysis of lymphocyte-mediated responses to HBV. Although the model cannot be extrapolated directly to immunocompetent hosts, it uniquely isolates hepatocyte-intrinsic fibrogenic mechanisms relevant to immunosuppressed HBV patients—a population with accelerated fibrosis risk. Future iterations incorporating human immune reconstitution could elucidate how metabolic changes in hepatocytes influence antiviral immunity by integration of multi-omics approaches for metabolomic, lipidomic and transcriptomic analyses. Spatial transcriptomics would help map the anatomical distribution of TIMP1-expressing bystander cells relative to infected foci, testing our hypothesis of paracrine signaling gradients. A critical limitation of this study is the absence of functional validation to delineate whether TIMP1 drives fibrosis initiation or merely reflects a secondary response to hepatic injury. Future studies should employ TIMP1 knockout or overexpression models are needed to establish causality between bystander HBV-uninfected hepatocytes and collagen deposition. Finally, the clinical correlation, while statistically significant, requires validation in larger longitudinal cohorts to assess TIMP1’s prognostic value. By dissecting HBV-host interactions at cellular and molecular levels, this work redefines our understanding of viral hepatitis pathogenesis.

In conclusion, our study offers valuable insights into the molecular mechanisms of the host-virus interactions and pathogenesis of chronic HBV infection and liver fibrosis initiation at the single cellular resolution with holistic transcriptional landscapes and metabolic reprogramming analysis. The finding that bystander hepatocytes contribute to fibrosis via TIMP1 rewiring shifts the focus of therapy from just targeting infected cells to modifying the overall hepatic microenvironment. Additionally, the metabolic weaknesses in HBV-infected hepatocytes offer potential targets for antiviral strategies. Novel avenues designed for therapeutic to disrupt viral persistence and fibrosis progression could be hence rationally derived. This study, the first to integrate humanized liver models with single-nucleus transcriptomics in HBV research, provides a framework for exploring host-pathogen interactions in other liver infections, enhancing our understanding of virology and informing precision medicine for chronic liver disease.

## Data Availability

The original contributions presented in the study are publicly available. This data can be found here: [https://ngdc.cncb.ac.cn/sso/loginservice=https://ngdc.cncb.ac.cn/gsa-human/login/ accession number: CRA025748].

## Supplementary material

Supplementary Figure 1 IF staining of Ki67 in URG, Hu-URG and HBV-hu-URG mice. Images of hTIMP1 (red), Ki67 (green), and Hoechst (blue) (Scale bars = 25 μm).

## References

[B1] Alvarez M. Benhammou J. N. Darci-Maher N. French S. W. Han S. B. Sinsheimer J. S.. (2022). Human liver single nucleus and single cell RNA sequencing identify a hepatocellular carcinoma-associated cell-type affecting survival. Genome Med. 14, 50. doi: 10.1186/s13073-022-01055-5, PMID: 35581624 PMC9115949

[B2] Andrews T. S. Atif J. Liu J. C. Perciani C. T. Ma X. Z. Thoeni C.. (2022). Single-cell, single-nucleus, and spatial RNA sequencing of the human liver identifies cholangiocyte and mesenchymal heterogeneity. Hepatol. Commun. 6, 821–840. doi: 10.1002/hep4.1854, PMID: 34792289 PMC8948611

[B3] Arthur M. J. (2000). Fibrogenesis II. Metalloproteinases and their inhibitors in liver fibrosis. Am. J. Physiol. Gastrointest Liver Physiol. 279, G245–G249. doi: 10.1152/ajpgi.2000.279.2.G245, PMID: 10915630

[B4] Barba G. Harper F. Harada T. Kohara M. Goulinet S. Matsuura Y.. (1997). Hepatitis C virus core protein shows a cytoplasmic localization and associates to cellular lipid storage droplets. Proc. Natl. Acad. Sci. U.S.A. 94, 1200–1205. doi: 10.1073/pnas.94.4.1200, PMID: 9037030 PMC19768

[B5] Bar-Yishay I. Shaul Y. Shlomai A. (2011). Hepatocyte metabolic signalling pathways and regulation of hepatitis B virus expression. Liver Int. 31, 282–290. doi: 10.1111/j.1478-3231.2010.02423.x, PMID: 21281428

[B6] Carlessi R. Denisenko E. Boslem E. Köhn-Gaone J. Main N. Abu Bakar N. D. B.. (2023). Single-nucleus RNA sequencing of pre-malignant liver reveals disease-associated hepatocyte state with HCC prognostic potential. Cell Genomics 3, 100301. doi: 10.1016/j.xgen.2023.100301, PMID: 37228755 PMC10203275

[B7] Cicchini C. Amicone L. Alonzi T. Marchetti A. Mancone C. Tripodi M. (2014). Molecular mechanisms controlling the phenotype and the EMT/MET dynamics of hepatocyte. Liver Int. 35, 302–310. doi: 10.1111/liv.12577, PMID: 24766136 PMC4344819

[B8] Cui F. Blach S. Manzengo Mingiedi C. Gonzalez M. A. Sabry Alaama A. Mozalevskis A.. (2023). Global reporting of progress towards elimination of hepatitis B and hepatitis C. Lancet Gastroenterol. Hepatol. 8, 332–342. doi: 10.1016/s2468-1253(22)00386-7, PMID: 36764320

[B9] Denisenko E. Guo B. B. Jones M. Hou R. de Kock L. Lassmann T.. (2020). Systematic assessment of tissue dissociation and storage biases in single-cell and single-nucleus RNA-seq workflows. Genome Biol. 21, 130. doi: 10.1186/s13059-020-02048-6, PMID: 32487174 PMC7265231

[B10] Diaz O. Vidalain P.-O. Ramière C. Lotteau V. Perrin-Cocon L. (2022). What role for cellular metabolism in the control of hepatitis viruses? Front. Immunol. 13. doi: 10.3389/fimmu.2022.1033314, PMID: 36466918 PMC9713817

[B11] Gao R. Kim C. Sei E. Foukakis T. Crosetto N. Chan L.-K.. (2017). Nanogrid single-nucleus RNA sequencing reveals phenotypic diversity in breast cancer. Nat. Commun. 8, 228. doi: 10.1038/s41467-017-00244-w, PMID: 28794488 PMC5550415

[B12] Guo G. H. Tan D. M. Zhu P. A. Liu F. (2009). Hepatitis B virus X protein promotes proliferation and upregulates TGF-beta1 and CTGF in human hepatic stellate cell line, LX-2. Hepatobiliary Pancreat Dis. Int. 8, 59–64., PMID: 19208517

[B13] Huang J. Zhao L. Yang P. Chen Z. Ruan X. Z. Huang A.. (2017). Fatty acid translocase promoted hepatitis B virus replication by upregulating the levels of hepatic cytosolic calcium. Exp. Cell Res. 358, 360–368. doi: 10.1016/j.yexcr.2017.07.012, PMID: 28697919

[B14] Jin Z. Sun R. Wei H. Gao X. Chen Y. Tian Z. (2011). Accelerated liver fibrosis in hepatitis B virus transgenic mice: involvement of natural killer T cells. Hepatology 53, 219–229. doi: 10.1002/hep.23983, PMID: 21140473

[B15] Kim K. H. Shin H. J. Kim K. Choi H. M. Rhee S. H. Moon H. B.. (2007). Hepatitis B virus X protein induces hepatic steatosis via transcriptional activation of SREBP1 and PPARgamma. Gastroenterology 132, 1955–1967. doi: 10.1053/j.gastro.2007.03.039, PMID: 17484888

[B16] Lai F. Wee C. Y. Y. Chen Q. (2021). Establishment of humanized mice for the study of HBV. Front. Immunol. 12. doi: 10.3389/fimmu.2021.638447, PMID: 33679796 PMC7933441

[B17] Lamontagne R. J. Casciano J. C. Bouchard M. J. (2018). A broad investigation of the HBV-mediated changes to primary hepatocyte physiology reveals HBV significantly alters metabolic pathways. Metabolism 83, 50–59. doi: 10.1016/j.metabol.2018.01.007, PMID: 29410347 PMC5960616

[B18] Li W. Duan X. Zhu C. Liu X. Jeyarajan A. J. Xu M.. (2022). Hepatitis B and hepatitis C virus infection promote liver fibrogenesis through a TGF-beta1-induced OCT4/nanog pathway. J. Immunol. 208, 672–684. doi: 10.4049/jimmunol.2001453, PMID: 35022275 PMC8770612

[B19] Li W. Yu X. Chen X. Wang Z. Yin M. Zhao Z.. (2020). HBV induces liver fibrosis via the TGF−β1/miR−21−5p pathway. Exp. Ther. Med. 21, 169. doi: 10.3892/etm.2020.9600, PMID: 33456536 PMC7792493

[B20] Lok A. S. F. (2024). Toward a functional cure for hepatitis B. Gut Liver 18, 593–601. doi: 10.5009/gnl240023, PMID: 38533651 PMC11249939

[B21] Masuzaki R. Kanda T. Sasaki R. Matsumoto N. Ogawa M. Matsuoka S.. (2020). Noninvasive assessment of liver fibrosis: current and future clinical and molecular perspectives. Int. J. Mol. Sci. 21, 4906. doi: 10.3390/ijms21144906, PMID: 32664553 PMC7402287

[B22] Miquilena-Colina M. E. Lima-Cabello E. Sanchez-Campos S. Garcia-Mediavilla M. V. Fernandez-Bermejo M. Lozano-Rodriguez T.. (2011). Hepatic fatty acid translocase CD36 upregulation is associated with insulin resistance, hyperinsulinaemia and increased steatosis in non-alcoholic steatohepatitis and chronic hepatitis C. Gut 60, 1394–1402. doi: 10.1136/gut.2010.222844, PMID: 21270117

[B23] Naim A. Pan Q. Baig M. S. (2017). Matrix metalloproteinases (MMPs) in liver diseases. J. Clin. Exp. Hepatol. 7, 367–372. doi: 10.1016/j.jceh.2017.09.004, PMID: 29234202 PMC5715451

[B24] Oh J.-M. An M. Son D.-S. Choi J. Cho Y. B. Yoo C. E.. (2022). Comparison of cell type distribution between single-cell and single-nucleus RNA sequencing: enrichment of adherent cell types in single-nucleus RNA sequencing. Exp. Mol. Med. 54, 2128–2134. doi: 10.1038/s12276-022-00892-z, PMID: 36460793 PMC9794763

[B25] Park S. A. Kim M. J. Park S. Y. Kim J. S. Lim W. Nam J. S.. (2015). TIMP-1 mediates TGF-beta-dependent crosstalk between hepatic stellate and cancer cells via FAK signaling. Sci. Rep. 5, 16492. doi: 10.1038/srep16492, PMID: 26549110 PMC4637930

[B26] Rouleau M. Schwab M. Klein K. Tremmel R. Haag M. Schaeffeler E.. (2025). The liver proteome of individuals with a natural UGT2B17 complete deficiency. Sci. Rep. 15, 5458. doi: 10.1038/s41598-025-89160-4, PMID: 39953065 PMC11828848

[B27] Song X. Gao X. Wang Y. Raja R. Zhang Y. Yang S.. (2021). HCV core protein induces chemokine CCL2 and CXCL10 expression through NF-kappaB signaling pathway in macrophages. Front. Immunol. 12. doi: 10.3389/fimmu.2021.654998, PMID: 34531848 PMC8438213

[B28] Song X. Guo Y. Duo S. Che J. Wu C. Ochiya T.. (2009). A mouse model of inducible liver injury caused by tet-on regulated urokinase for studies of hepatocyte transplantation. Am. J. Pathol. 175, 1975–1983. doi: 10.2353/ajpath.2009.090349, PMID: 19808649 PMC2774061

[B29] Vogt D. A. Camus G. Herker E. Webster B. R. Tsou C. L. Greene W. C.. (2013). Lipid droplet-binding protein TIP47 regulates hepatitis C Virus RNA replication through interaction with the viral NS5A protein. PloS Pathog. 9, e1003302. doi: 10.1371/journal.ppat.1003302, PMID: 23593007 PMC3623766

[B30] Wang Y. Wu T. Hu D. Weng X. Wang X. Chen P. J.. (2018). Intracellular hepatitis B virus increases hepatic cholesterol deposition in alcoholic fatty liver via hepatitis B core protein. J. Lipid Res. 59, 58–68. doi: 10.1194/jlr.M079533, PMID: 29133292 PMC5748497

[B31] Wang H. Zhang J. (2023). The glucose metabolic reprogramming in hepatitis B virus infection and hepatitis B virus associated diseases. J. Gastroenterol. Hepatol. 38, 1886–1891. doi: 10.1111/jgh.16340, PMID: 37654246

[B32] Wen F. Tang X. Xu L. Qu H. (2022). Comparison of single−nucleus and single−cell transcriptomes in hepatocellular carcinoma tissue. Mol. Med. Rep. 26, 339. doi: 10.3892/mmr.2022.12855, PMID: 36111491 PMC9494604

[B33] Wu H. Kirita Y. Donnelly E. L. Humphreys B. D. (2019). Advantages of single-nucleus over single-cell RNA sequencing of adult kidney: rare cell types and novel cell states revealed in fibrosis. J. Am. Soc. Nephrol. 30, 23–32. doi: 10.1681/asn.2018090912, PMID: 30510133 PMC6317600

[B34] Xie B. Sun D. Du Y. Jia J. Sun S. Xu J.. (2019). A two-step lineage reprogramming strategy to generate functionally competent human hepatocytes from fibroblasts. Cell Res. 29, 696–710. doi: 10.1038/s41422-019-0196-x, PMID: 31270412 PMC6796870

[B35] Yasumoto J. Kasai H. Yoshimura K. Otoguro T. Watashi K. Wakita T.. (2017). Hepatitis B virus prevents excessive viral production via reduction of cell death-inducing DFF45-like effectors. J. Gen. Virol. 98, 1762–1773. doi: 10.1099/jgv.0.000813, PMID: 28745269

[B36] Zan Y. Zhang Y. Tien P. (2013). Hepatitis B virus e antigen induces activation of rat hepatic stellate cells. Biochem. Biophys. Res. Commun. 435, 391–396. doi: 10.1016/j.bbrc.2013.04.098, PMID: 23665329

[B37] Zeisberg M. Yang C. Martino M. Duncan M. B. Rieder F. Tanjore H.. (2007). Fibroblasts derive from hepatocytes in liver fibrosis via epithelial to mesenchymal transition. J. Biol. Chem. 282, 23337–23347. doi: 10.1074/jbc.M700194200, PMID: 17562716

[B38] Zeng W. Jiang S. Kong X. El-Ali N. Ball A. R. Ma C. I. H.. (2016). Single-nucleus RNA-seq of differentiating human myoblasts reveals the extent of fate heterogeneity. Nucleic Acids Res. 44, e158. doi: 10.1093/nar/gkw739, PMID: 27566152 PMC5137429

[B39] Zhang J. Ling N. Lei Y. Peng M. Hu P. Chen M. (2021). Multifaceted interaction between hepatitis B virus infection and lipid metabolism in hepatocytes: A potential target of antiviral therapy for chronic hepatitis B. Front. Microbiol. 12. doi: 10.3389/fmicb.2021.636897, PMID: 33776969 PMC7991784

[B40] Zhou L. He R. Fang P. Li M. Yu H. Wang Q.. (2021). Hepatitis B virus rigs the cellular metabolome to avoid innate immune recognition. Nat. Commun. 12, 98. doi: 10.1038/s41467-020-20316-8, PMID: 33397935 PMC7782485

[B41] Zhu C. L. Li W. T. Li Y. Gao R. T. (2012). Serum levels of tissue inhibitor of metalloproteinase-1 are correlated with liver fibrosis in patients with chronic hepatitis B. J. Dig Dis. 13, 558–563. doi: 10.1111/j.1751-2980.2012.00629.x, PMID: 23107442

