# Supplementary figures and images for: Single-nucleus RNA sequencing reveals HBV-driven metabolic reprogramming and TIMP1-mediated fibrosis in human-liver-chimeric mice
Source: Front Cell Infect Microbiol. 2025 Sep 2;15:1654903. doi: 10.3389/fcimb.2025.1654903 (PMC12436295; doi:10.3389/fcimb.2025.1654903)

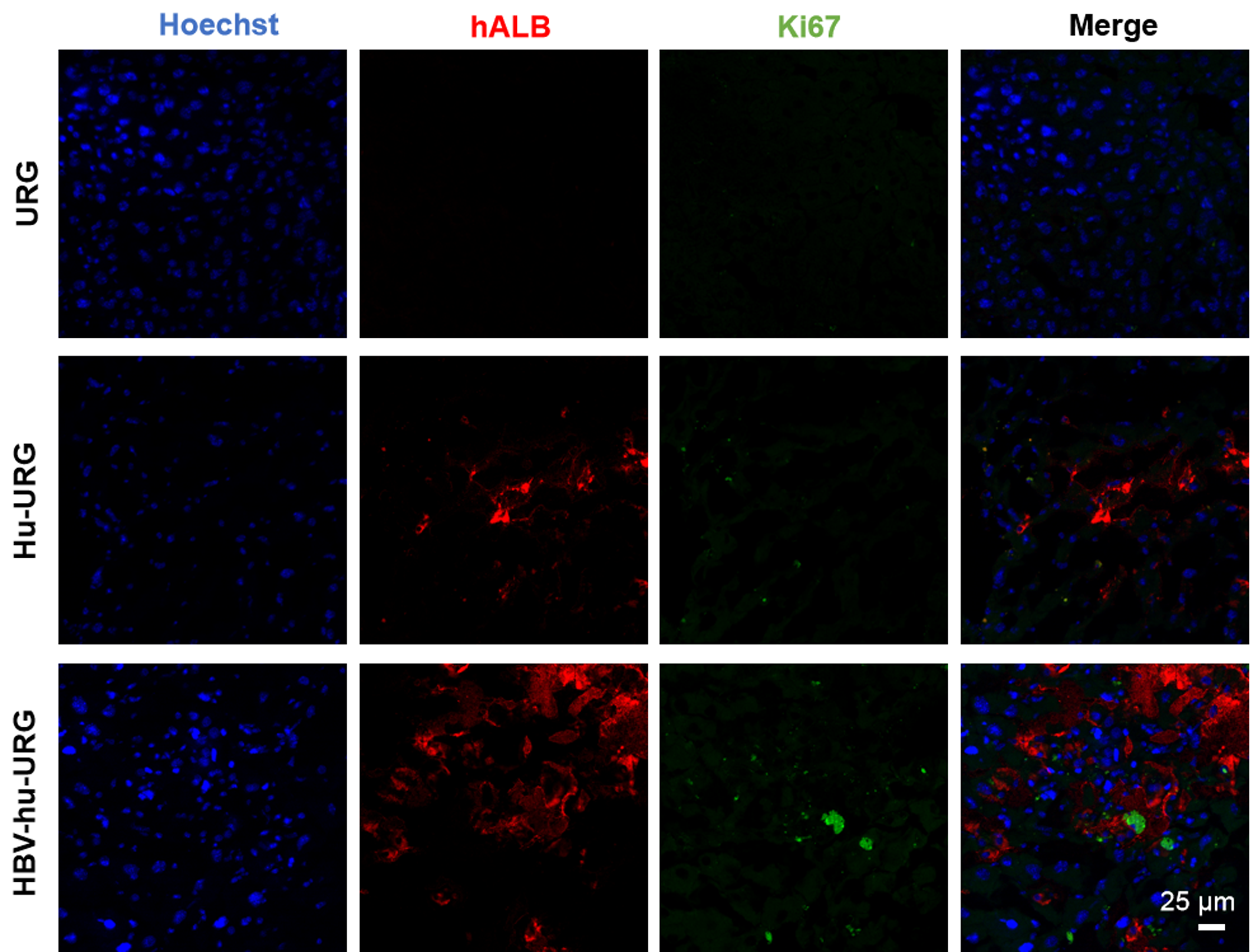

Supplement: Supplementary Figure 1 — IF staining of Ki67 in URG, Hu-URG and HBV-hu-URG mice. Images of hTIMP1 (red), Ki67 (green), and Hoechst (blue) (Scale bars = 25 μm). [file Image1.tif]
